# Non-pharmacological therapies for patients with lymphangioleiomyomatosis: a systematic review and meta-analysis

**DOI:** 10.3389/fresc.2026.1819549

**Published:** 2026-06-02

**Authors:** Yingwen Yu, Fang Lei, Huizi Wang

**Affiliations:** 1 First Affiliated Hospital of Guangzhou Medical University, Guangzhou, China; 2 School of Nursing, University of Minnesota Twin Cities, Minneapolis, MN, United States

**Keywords:** 6-minute walking distance test, lymphangioleiomyomatosis, non-pharmacological therapy, physical activity tolerance, pulmonary function, quality of life

## Abstract

**Objectives:**

This study aims to systematically review non-pharmacological therapies for patients with lymphangioleiomyomatosis (LAM), and synthesize the effects of non-pharmacological therapies on 6-minute walking distance test (6MWDT), pulmonary function (FEV₁), and quality of life in LAM patients.

**Methods:**

The systematic review and meta-analysis design was used. Data were searched on Scopus, Web of Science, PubMed, Embase, OVID, CINAHL, CNKI and Wang Fang for studies published from August 2015 to August 2025 on non-pharmacological therapies for patients with LAM. Studies were screened using rigorous inclusion and exclusion criteria. Study quality was assessed using JBI study quality evaluation tools, and a stratified narrative synthesis was conducted based on intervention types, supplemented with a meta-analysis (excluding single-case report).

**Results:**

Five studies were eligible to be included in this study. Narrative synthesis showed that interventions with multicomponent, including high intensity aerobic and strength training, yoga, pulmonary rehabilitation and telemonitoring exercise, are beneficial for patients with LAM. Meta-analysis results (excluding the case report) showed that non-pharmacological therapies, particularly structured exercise programs, significantly increased physical activity tolerance of patients with LAM (*p* < 0.00001) by 37.39 meters using 6MWDT, compared to the pre-intervention or comparison group (95% CI: 27.29, 46.98). Non-pharmacological therapies increased pulmonary function FEV₁ (MD: 0.20, 95% CI: −0.02, 0.42) and quality of life (SMD: −0.19; 95%CI: −0.56, 0.18) of the patients with LAM, compared to the pre-intervention or comparison group, however, the results were not significant (*p* = 0.08 and 0.32, respectively). No serious adverse events were reported in the included studies, indicating good safety of non-pharmacological interventions.

**Discussion:**

Non-pharmacological therapies, especially physiotherapy, can improve patients with LAM's physical activity tolerance, enhancing their physical fitness and daily activity capacity. Compared with other alveolar and parenchymal diseases (e.g., tuberculosis, COPD), LAM patients show similar improvements in exercise tolerance but less significant improvements in lung function, which may be related to the unique cystic lung pathology of LAM. More effectively and innovatively designed research is needed to improve patients with LAM's pulmonary function and quality of life.

**Conclusions:**

This study not only demonstrates the potential value of non-pharmacological therapies in the management of patients with LAM, but also points the directions for future research. By further optimizing the interventions and expanding the sample size, we can achieve a comprehensive understanding of the long-term effects of non-pharmacological therapies in patients with LAM, eventually providing more effective treatment options for patients.

**Systematic Review Registration:**

https://www.crd.york.ac.uk/prospero/, PROSPERO system ID: CRD42024548486.

## Contribution of the paper

- Interventions with multicomponent, including high intensity aerobic and strength training, yoga, pulmonary rehabilitation and telemonitoring exercise, are beneficial and safe for patients with LAM.
- Non-pharmacological therapies, particularly structured exercise programs, significantly enhance physical activity tolerance (measured by 6MWDT) in patients with LAM. However, the impact on pulmonary function (FEV₁) and quality of life remains less conclusive.
- This study provides evidence for clinical management of patients with LAM, and compares the effects of non-pharmacological therapies with other alveolar and parenchymal diseases, offering a broader perspective.

## Introduction

Lymphangioleiomyomatosis (LAM) is a progressive pulmonary systemic disease. It is considered as a low malignant tumor characterized by the atypical smooth muscle cell abnormally growing in pulmonary vasculature, lymphatic vessels, and alveoli (1). LAM causes formation of multiple cysts in bilateral lungs and appears outside of kidney, causing benign renal angiomyolipomas and perivascular epithelioid cell tumor involving multiple internal organs (e.g., liver, spleen, and lymph nodes) (1).

LAM often occurs in women of childbearing age. It is mainly divided into two categories: sporadic LAM (S-LAM) and tuberous-sclerosis-related LAM (TSC-LAM). It was first reported by Lutembacher in 1918 and described as cystic lung disease related to TSC (2). In 1937, Von Stossel reported the first case of S-LAM (3). The specific cause of S-LAM is unknown, while it is reported that TSC is a genetic disease-causing benign tumor which can occur in multiple organs throughout the body (e.g., the brain, skin, kidney, and heart) (4).

### Epidemiology

LAM has been reported worldwide, but the incidence rates vary among regions due to its rarity. Several studies about LAM have been done in developed countries such as the United States, Japan, and European countries. It is reported that the incidence of LAM is estimated at 3 to 8 cases per million people (5). One study conducted in seven countries estimated that three to seven cases of S-LAM occur per million women (6). Another study showed that one in 400,000 adult women had S-LAM (5). TSC-LAM is more common than S-LAM, with about 30 to 40% of patients with TSC developing LAM. For instance, a study reported that approximately 32% of female patients with TSC were diagnosed with LAM (7).

According to the results of several studies, the 5-year survival rate of patients with LAM is about 86% to 93%, and the 10-year survival rate is between 80% and 90% (8). Specifically, the 5-year transplant-free survival rate is approximately 93% for patients with LAM, and the 10-year transplant-free survival rate is 86% (9).

### Symptoms and progression

LAM is characterized by progressive worsening dyspnea and recurrent pneumothorax and chylothorax (10). Once a patient develops pneumothorax, the chance of recurrence is greater than 70%. All patients with LAM should be informed of the risk of pneumothorax. Chemical pleurodesis treatment can be considered for patients who are diagnosed with pneumothorax for the first time (10).

Patients with LAM often have a change in their lung functions. The initial assessments should include pulmonary ventilatory function test, bronchodilation test, and diffusion function test (11). The forced expiratory volume in one second (FEV₁) and diffusing capacity of the lungs for carbon monoxide (DLCO) are associated with chest Hampton Roads Communication Technologies (HRCT) result and histological abnormalities (e.g., signs and symptoms) in LAM disease, which change accordingly with the progression of LAM (5). The severity and progression of LAM can be assessed by the pathophysiological assessment score of lungs, quantification of the computed tomography result, lung function tests, and 6-minute walking distance test (6MWDT) (11).

### Therapeutic methods

Due to the complex pathological mechanisms and multi-system effects of LAM, treatment of LAM requires both pharmacological and non-pharmacological approaches. The combination of non-pharmacological and pharmacological therapies helps patients to achieve the best treatment effect by slowing down the progress of disease, preventing complications, and controlling disease comprehensively.

However, for the patients with end-stage LAM (whose cardiac function is at level III or IV according to the New York Heart Association cardiac function classification, having a low oxygen saturation rate at rest and severe impairment of lung function and physical activity tolerance), if the absence of effective therapy ultimately leads to respiratory insufficiency, lung transplantation is the only effective treatment (5).

#### Pharmacological therapies

LAM is considered as a low-grade malignancy related to cancer. It is caused by mutations in the TSC 1/2 genes resulting in constitutive activation of the kinase mammalian target of rapamycin (mTOR) (5).

Although the prognosis of LAM varies by individual variation, patient survival has been significantly longer in recent years with advances in diagnostic techniques and improved treatments. Pharmacotherapy drugs targeting signaling pathways are thought to be important in disease pathogenesis. The focus of pharmacotherapy for LAM includes relieving symptoms and slowing disease progression.

Currently, the most commonly used drugs include mTOR inhibitors and hormonal drugs (11, 12). Sirolimus and everolimus are two mTOR inhibitors which have been shown to be effective in stabilizing lung function and reducing the size of chylous effusion, lymphangioleiomyoma, and alleviating renal angiomyolipoma (AML) (11, 12). In some cases, hormonal drugs are also used in the treatment of LAM, especially in the control of acute symptoms. For example, corticosteroids (e. g., prednisone) can be used to reduce inflammation and relieve acute dyspnea. However, long-term use of hormone drugs can cause side effects, so they are usually only used as adjuvant therapy or short-term relieve (13).

In addition, there is evidence that increased serum levels of vascular endothelial growth factor D (VEGF-D) in patients with LAM are associated with disease severity and clinical course (14). Blocking Vascular Endothelial Growth Factor Receptor (VEGFR) or anti-VEGF therapy is considered as an effective way to treat LAM (14). But it has not yet become standard treatment.

Furthermore, bronchodilators can also be used in some patients with LAM, although their effect is usually limited. Bronchodilators can help relieve airway obstruction and improve dyspnea. These drugs can be used as part of supportive care to help manage patients' respiratory symptoms and improve their quality of life (4).

#### Non-pharmacological therapies

In the management of LAM, non-pharmacological therapies play a crucial role, not only helping to improve symptoms of the disease but also delaying the progression of the disease. Non-pharmacological treatments such as surgical therapy, pulmonary rehabilitation, oxygen therapy, psychological counseling, and nutritional support are important in patients with LAM, and each plays a unique role in improving patient outcomes. For patients with recurrent pneumothorax, chemical pleurodesis or surgical pleurectomy can be performed to reduce the risk of recurrence; for end-stage LAM patients with severe respiratory insufficiency, lung transplantation is the only effective treatment to improve survival and quality of life (10). As a core component of non-pharmacological therapies in patients with LAM, pulmonary rehabilitation can significantly improve physical activity tolerance and respiratory function through systematic aerobic exercise and strength training (5). It includes exercise training, respiratory training, and patient education, helping patients master self-management skills. For LAM patients with hypoxemia at rest or during exercise, long-term oxygen therapy can effectively relieve dyspnea and fatigue, improve tissue oxygenation, and enhance exercise tolerance (13). In addition, LAM is a chronic progressive disease that often causes anxiety, depression, and other psychological problems in patients. Psychological support and counseling help patients cope with stress from the illness, improve psychological resilience, and enhance treatment adherence, thereby improving overall quality of life (4). Lastly, proper nutritional support helps control the symptoms of LAM and slow the progression. For example, a low-fat diet and medium chain triglycerides (MCT) can reduce the production and accumulation of chylous fluid, which is beneficial for patients with chylothorax or chylous ascites (4).

Although pharmacological therapies of LAM have been systematically reported and analyzed (12, 15), and non-pharmacological therapies of LAM have shown their value to some extent, there is still a lack of targeted synthesis of 6MWDT outcomes and comprehensive review of all non-pharmacological interventions. Synthesizing non-pharmacological therapies of LAM and focusing on 6MWDT outcomes can further improve the strategies of non-drug treatment for patients with LAM and guide to improve their quality of life and disease management effectiveness.

### Purpose

This study aims to 1) systematically review non-pharmacological therapies for patients with LAM, with a focus on surgical therapy, pulmonary rehabilitation, oxygen therapy, psychological counseling, and nutritional support; and 2) synthesize the effects of non-pharmacological therapies on 6MWDT, pulmonary function (FEV₁), and quality of life in LAM patients, to provide evidence-based support for clinical management.

This study will help to drive more diverse and comprehensive non-pharmacological intervention studies by identifying gaps and deficiencies in existing research. By emphasizing the contribution of non-pharmacological therapies to improve outcomes of patients with LAM, this study helps health care providers to understand and apply non-pharmacological treatments, prompt patients to actively participate in these treatments, and ultimately improve patients' long-term outcomes.

## Materials and methods

### Design

This study is a systematic review and meta-analysis study. The study protocol was registered in the PROSPERO system (ID: CRD42024548486). The review protocol can be accessed on the PROSPERO website (https://www.crd.york.ac.uk/prospero/). No amendments were made to the review protocol.

### Data sources and search strategies

We systematically searched the Scopus, Web of Science, PubMed, Embase, OVID, CINAHL, CNKI, and WANGFANG databases for studies published from August 2015 to August 2025 on non-pharmacological therapies for patients with LAM. Given the rarity of LAM and the anticipated scarcity of literature specifically addressing treatment, we intentionally expanded our search scope by focusing primarily on the Population (P) and Intervention (I) elements of the PICO framework. Consequently, elements for Comparison (C) and Outcomes (O) were not included in the formal search strategies to maximize retrieval of potentially relevant studies. To further reduce the risk of omitting eligible studies, we conducted a grey literature search using Google Scholar and examined relevant conference proceedings where available. Additionally, we employed snowballing techniques by screening the reference lists of included articles and relevant reviews to identify any additional studies.

The search strategies incorporated both controlled vocabulary and free-text terms. Specifically, we used Medical Subject Headings (MeSH, standardized subject descriptors within PubMed and the Health Sciences Virtual Health Library) and equivalent descriptors from other databases (e.g., Emtree in Embase) to enhance retrieval precision and indexing. For the population of interest, we applied MeSH terms such as “Lymphangioleiomyomatosis” and supplemented these with synonymous free-text terms (e.g., “Lymphangiomyomatosis,” “Lymphophangiomyomatosis”) to account for variations in terminology. For interventions, we used MeSH terms including “Rehabilitation,” “Physical Therapy Modalities,” “Oxygen Inhalation Therapy,” “Diet Therapy,” “Psychological Counseling”, and “Surgical Procedures, Operative”, along with relevant synonyms (e.g., “non-pharmacological,” “non-drug”). The term “Therapeutics” and related intervention terms were also included. This combined approach ensured a comprehensive and sensitive search strategy aimed at capturing all potentially relevant literature across multiple medical subject databases.

### Inclusion and exclusion criteria

Inclusion criteria for the studies were original interventional or comparative studies with defined non-pharmacological interventions, including randomized controlled trials (RCTs), non-randomized controlled trials, prospective or retrospective cohort studies with control groups, before-after studies with pre/post intervention data. Exclusion criteria were: 1) Studies focusing on other therapies (e.g., pharmacological therapies) of patients with LAM; 2) studies which were conducted in patients without LAM; 3) observational studies without intervention (e.g., cross-sectional studies, case-control studies); 4) secondary studies without primary data (e.g., systematic reviews, narrative reviews), 5) commentary, editorials, conference abstracts, or unpublished data.

### Rationale for study design inclusion

Controlled trials and longitudinal interventional designs were prioritized to establish causality between interventions and outcomes (16). Cross-sectional studies were excluded as they cannot demonstrate temporal relationships. One Case report (17) was included in the narrative synthesis of this study due to its unique contribution to high-intensity exercise safety data in LAM but was excluded from the meta-analysis to avoid bias from single-case data.

### Selection process

Two researchers (the first author and third author of this study) independently searched the literature. Eligible studies were imported to the NoteExpress and Zotero software, respectively. Duplicate articles were eliminated during the process. Titles and abstracts of all the remaining eligible articles were further checked by the two researchers. Subsequently, full texts of the selected articles were read for comprehensive evaluation.

### Data extraction

Two researchers (the first author and third author of this study) independently extracted data and entered data to the table of evidence. Disagreements were resolved by several discussions between them and extra rounds of consultation with another researcher (the corresponding author of this study). Data extracted from the studies included authors, year of publication, country of origin, sample size, intervention method, time/frequency/duration of the intervention, and study findings (focusing on 6MWDT, FEV₁, and quality of life).

### Quality assessment

Methodological quality was assessed using the following validated tools from the Joanna Briggs Institute (JBI) Critical Appraisal suite (18–20): 1) Quasi-experimental studies: JBI Checklist for Quasi-Experimental Studies (2020 version) (18); 2) retrospective studies: JBI Checklist for Analytical Cross-Sectional Studies (2020 version) (19); 3) case reports: JBI Checklist for Case Reports (2021 version) (20). Scoring systems were implemented per the developers' guidelines (18–20): 1) Quasi-experimental: 0–3 (low), 4–6 (moderate), 7–9 (high) (18); 2) retrospective: 0–4 (low), 5–7 (moderate), 8–10 (high) (19); 3) case reports: 0–2 (low), 3–5 (moderate), 6–8 (high) (20). These assessment tools ensure comprehensive understanding of the scientific nature and rigor of the studies, help to identify the strengths and weaknesses of each study, and provide a valuable reference for future research.

### Data analysis

The narrative evidence was synthesized using the comparison data analysis, stratified by intervention type (high-intensity training, pulmonary rehabilitation, yoga, telemonitoring exercise). We performed the meta-analysis using Review Manager Version 5.4 software (21), excluding the single case report to ensure robustness. We used Tau **^2^** and I**^2^** to assess heterogeneity of the included studies and to reveal the variance across studies (22). The I**^2^** was categorized as no heterogeneity (0% −25%), low heterogeneity (25% -50%), medium heterogeneity (50% -75%), and high heterogeneity (75% -100%) (23). Since these studies used different measurement scales to measure patients' quality of life, we used the standardized mean differences and 95% confidence intervals to measure the estimated effect size on patients' quality of life (24). Some of the included studies were quasi-experimental study without control group, so the pre-intervention data were used as a comparison to evaluate the effect of the intervention (25). Effect sizes and normalized mean differences for 95% CI were visualized as forest plots (26). In addition, we assessed the risk of publication bias of the included studies following the PRISMA recommendations. Publication bias was visually checked using funnel plots. The first author performed the data analysis, and the fourth author reviewed and validated the results.

## Results

### Study selection

**Figure 1:**
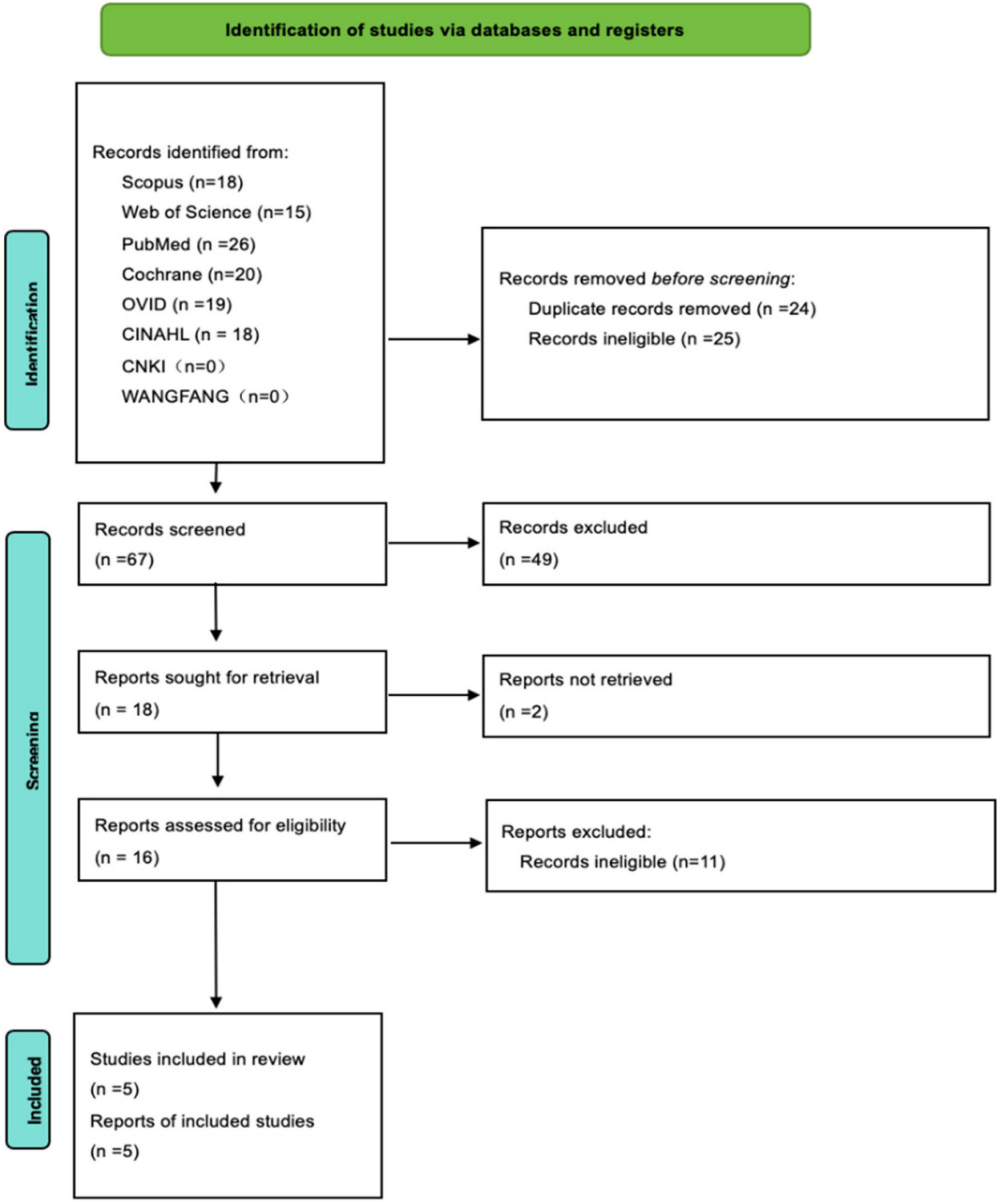
PRISMA flowchart.

Following the PRISMA 2020 guidelines (27) (Figure 1), a total of 116 records were identified through database searches. After removing duplicates (*n* = 24) and ineligible records (*n* = 25), 67 studies underwent title/abstract screening. Of these, 49 were excluded due to irrelevant population, intervention, or study design. The remaining 18 full-text articles were assessed, with 13 excluded for failing inclusion criteria (e.g., non-LAM population, pharmacological interventions; *n* = 11) or unavailability of full texts (*n* = 2). Five studies met all criteria and were included in the final analysis (Figure 1, Table 1).

**Table 1:** Characteristics of the included studies (*n* = 5).

Study	Design	Intervention Type	Key Intervention Components	Frequency & Duration	Delivery Mode
Lowder (17)	Case Report	High-Intensity Training	Aerobic (running/sprinting) + Resistance training	Frequency: 2x/week (60min/session) Duration: 1 year	Supervised
Araujo et al. (28)	Quasi-experimental	Pulmonary Rehabilitation (PR)	Aerobic (treadmill) + Strength training + Education	Frequency: 2x/week (1h/session) Duration: 24 weeks	Supervised, Outpatient
Child et al. (29)	Quasi-experimental	Telemonitored Exercise	Aerobic + Strength training (remote monitoring)	Frequency: Aerobic 4x/week, Strength 3x/week Duration: 12 weeks	Remote, Home-based
Li et al. (30)	Quasi-experimental	Yoga + Rehabilitation	Yoga classes + Home practice + Aerobic/Strength training	Frequency: Class 1x/week (90min) + Home ≥2x/week (15min) Duration: 24 weeks	Supervised (class) + Home
Gloeckl et al. (31)	Retrospective Analysis	Inpatient Pulmonary Rehabilitation (PR)	Aerobic + Strength training + Education/Self-management	Frequency: 5–6 days/week Duration: 4 weeks	Supervised, Inpatient

### Study characteristics

The five articles varied in study design, including three quasi-experimental studies (28–30), one retrospective analysis study (31), and one case report (17). The publication year of the included studies ranged from 2016 to 2023. Purposes of the included studies were: To evaluate the effect of high-intensity aerobic training (17), pulmonary rehabilitation (28), Yoga (30), telemonitoring exercise (29), and inpatient pulmonary rehabilitation (31) in patients with LAM, respectively.

### Quality assessment outcomes

The methodological quality of included studies was rigorously assessed using Joanna Briggs Institute (JBI) critical appraisal tools. Detailed item-by-item ratings are presented in Table 2.

**Table 2:** Methodological quality assessment of included studies using JBI critical appraisal tools.

Study	Design	JBI Checklist Items (Score/Total)	Overall Rating	Key Weaknesses
Lowder (17)	Case report	7/8	High	Single-case design
Araujo et al. (28)	Quasi-experimental	8/9	High	No randomization
Child et al. (29)	Quasi-experimental	5/9	Moderate	No control group
Li et al. (30)	Quasi-experimental	7/9	High	Small sample size
Gloeckl et al. (31)	Retrospective analysis	9/10	High	Potential recall bias

Footnotes: JBI Tools: Quasi-experimental: 9 items (e.g., cause-effect clarity, baseline comparability); Retrospective, 10 items (e.g., confounder adjustment, outcome measurement); Case report, 8 items (e.g., patient history, intervention description). Scoring: Y = Yes (1 point), *N* = No (0 point).

As shown in Table 2, three studies (18, 29, 32) met over 85% of JBI criteria, indicating robust methodology. Common limitations included small sample sizes (30) and lack of randomization (29). No studies were excluded based on quality thresholds, but these factors were considered in evidence synthesis.

#### Quasi-experimental studies (*n* = 3)

Araujo et al. (26) and Li et al. (30) met almost all JBI criteria, demonstrating comprehensive design and reliable results. Child et al. (29) scored lower due to the absence of a control group, potentially impacting validity.

#### Retrospective analysis

Gloeckl et al. (31) achieved high quality, fulfilling almost all JBI criteria for retrospective studies. Strengths included robust adjustment for confounders, reliable outcome measures, and appropriate statistical methods.

#### Case report

Lowder (17) met almost all JBI case report criteria. The study provided clear, detailed methodology and outcomes with no reported adverse events.

#### Overall rigor

All studies implemented stringent participant screening and enrollment criteria, enhancing internal validity. Collectively, these assessments support the utility of the included evidence for evaluating non-pharmacological therapies in LAM.

### Evidence synthesis

In the included studies, non-pharmacological therapies for patients with LAM included high intensity aerobic and strength training, yoga, pulmonary rehabilitation and telemonitoring exercise. For example, Lowder (17) provided high-intensity aerobic and strength training to patients twice a week, primarily through running and resistance training. Li et al. (30) conducted a 24-week yoga course with 90-minute per session plus home practice no less than twice a week. Araujo et al. (28) implemented the pulmonary rehabilitation program which included 60 min of training twice a week, with 30 min of treadmill aerobic exercise and 30 min of muscle strength training. Child et al. (29) conducted a 12-week telemonitoring exercise program with four aerobic sessions of 25 to 45 min per week, combined with three strength training sessions. No study reported the surgical therapy, psychological counseling, and nutritional support results of patients with LAM.

These studies varied in intervention frequency and duration, but all emphasized long-term sustained training. For example, the study conducted by Lowder 16 lasted for one year with twice weekly intensive training. The study conducted by Araujo et al. (28) lasted 24 weeks with twice weekly yoga. In Li et al.'s study (30), the intervention lasted for 24 weeks. And in Child et al. (29) study, the intervention lasted for 12 weeks with seven weekly exercises (four aerobic exercises and three strength trainings). The frequency and duration were carefully considered by the research teams to provide sufficient time and intensity to observe the intervention effects.

High adherence and retention rates were consistently observed across studies. Attendance rates exceeded 80% in structured interventions: 91.7% median attendance for yoga (30), over 80% completion for pulmonary rehabilitation (28), and consistent task completion in telemonitoring exercise (29). These high adherence levels support the feasibility of non-pharmacological interventions in LAM management.

Safety of the interventions was generally reported across the studies, and no serious adverse events were identified. For example, to prevent exercise intolerance and safety issues, Child et al. (29) ensured all participants maintained an oxygen saturation of 85% during exercise in the telemonitoring exercise program. Li et al. (30) reported no yoga-related injuries or other major adverse events in the yoga group. Similarly, Gloeckl (31) reported no adverse events, supporting the safety of inpatient rehabilitation. Lowder (17) also found no serious adverse events happened during the program, indicating that the pulmonary rehabilitation program is feasible in terms of safety.

Table 3 showed the adverse events and dropout data in the included studies. In summary, dropout rates of the studies ranged from 0 to 14.3% (mean=5.7%). In the studies, only mild-to-moderate transient adverse events reported; no serious intervention-related adverse events occurred; and common reasons for dropout are personal scheduling conflicts (3/4) and unrelated health issues (1/4).

**Table 3:** Adverse events and dropout rates of the included studies.

Study	Intervention	Dropout rate (%)	Adverse Events Reported
Lowder (17)	High-intensity training	0/1 (0%)	Mild exertional dyspnea (resolved with rest)
Araujo et al. (28)	Pulmonary rehabilitation	1/19 (5.3%)	Muscle soreness: 2/18 (11.1%), Transient dyspnea: 1/18 (5.6%)
Child et al. (29)	Telemonitoring exercise	2/14 (14.3%)	Exercise-induced hypoxia (SpO₂<85%): 3 episodes in 2 patients
Li et al. (30)	Yoga + rehabilitation	1/11 (9.1%)	Yoga-related joint pain: 1/10 (10%)
Gloeckl et al. (31)	Inpatient PR	0/58 (0%)	None documented

### Effect of nonpharmacological therapy in patients with LAM

#### 6MWDT

**Figure 2:**
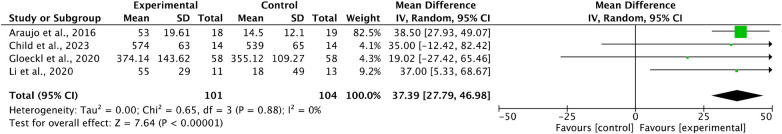
Effect of nonpharmacological therapy on 6MWD in patients with LAM.

Among the five included studies, four studies measured the outcome of 6MWD in patients with LAM (28–31). The meta-analysis result showed that non-pharmacological interventions improved patients' 6MWD. Compared to the control group or pre-intervention, patients with LAM in the intervention group had a significant increase (*p* < 0.00001) on their 6 MWD by 37.39 m (95% CI: 27.79, 46.98) after non-pharmacological intervention. The heterogeneity analysis showed no heterogeneity (Tau ^2^ = 0.00, ChI^2^ = 0.65, df = 3, *p* = 0.88, I^2^ = 0%), indicating high agreement across study findings (Figure 2).

#### Pulmonary function test FEV_1_

**Figure 3:**
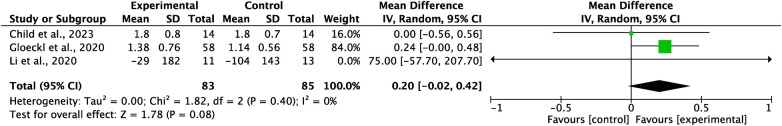
Effect of nonpharmacological therapy on pulmonary function test FEV_1_ in patients with LAM.

Among the five included studies, three studies measured the outcome of FEV_1_ in patients with LAM (excluding the single case report study (29–31). The meta-analysis result showed that non-pharmacological interventions improved patients' FEV_1_. Compared to the control group or pre-intervention, patients with LAM in the intervention group had an increase on their FEV_1_ by 0.20L (95% CI: −0.02, 0.42) after non-pharmacological intervention, however, the difference was not significant (*p* = 0.08). The heterogeneity analysis showed no heterogeneity (Tau^2^ = 0.00, ChI^2^ = 1.82, df = 2, *p* = 0.40, I^2^ = 0%), indicating high agreement across studies (Figure 3).

#### Quality of life

**Figure 4:**
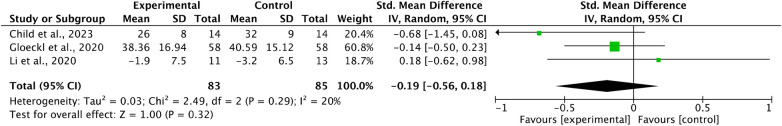
Effect of nonpharmacological therapy on quality of life in patients with LAM.

Among the five included studies, three studies measured the outcome of quality of life in patients with LAM (29–31). The meta-analysis result showed that non-pharmacological interventions improved patients' quality of life. Compared to the control group or pre-intervention, non-pharmacological therapies were associated with less decreased quality of life (mean difference, −0.19; 95% CI, −0.56, 0.18), however, the difference was not significant (*p* = 0.32). The heterogeneity analysis showed low heterogeneity (Tau^2^ = 0.03, Chi^2^ = 2.49, df = 2, *p* = 0.29, I^2^ = 20%), indicating high agreement across the study findings (Figure 4).

### Publication bias

**Figure 5:**
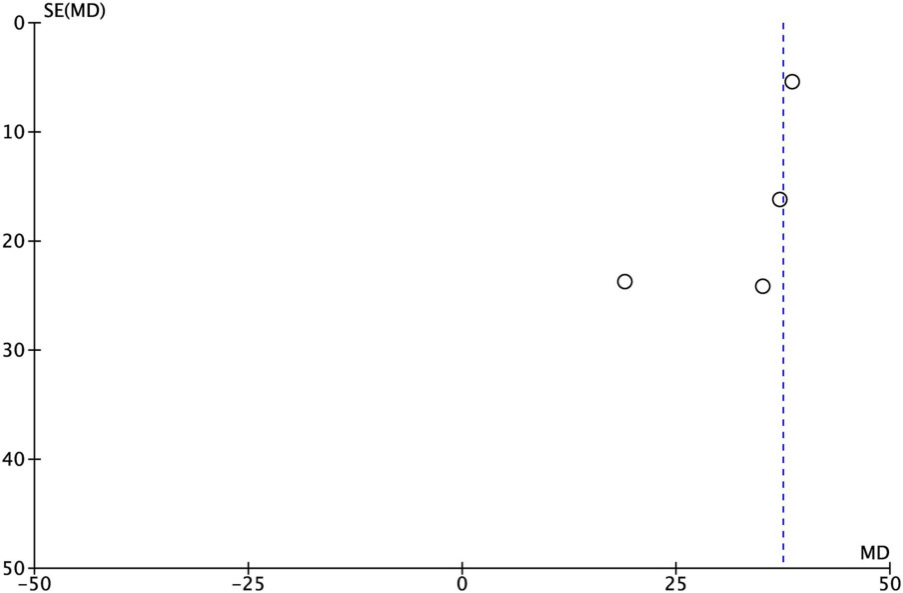
Funnel plot. Effect of nonpharmacological therapy on 6MWD in patients with LAM. SE: standard error, MD: mean difference.

**Figure 6:**
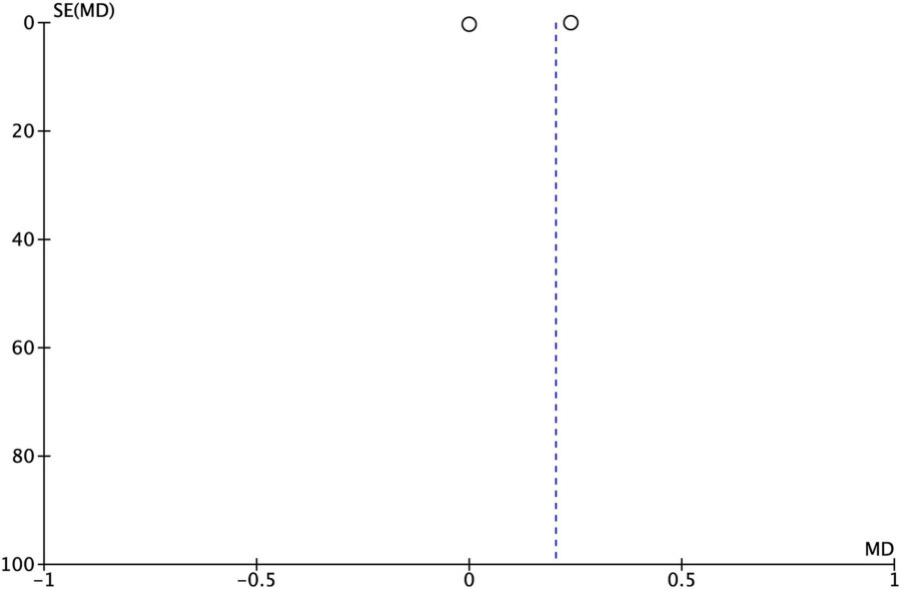
Funnel plot. Effect of nonpharmacological therapy on pulmonary function test FEV_1_ in patients with LAM. SE, standard error; MD, mean difference.

**Figure 7:**
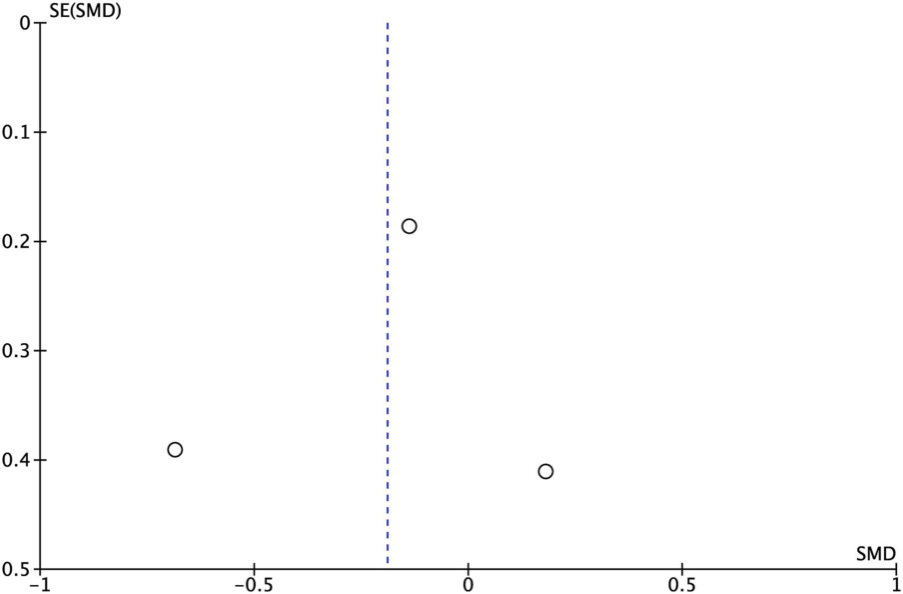
Funnel plot. Effect of nonpharmacological therapy on quality of life in patients with LAM. SE, standard error; SMD, standardized mean difference.

Funnel plots were generated respectively for each primary outcome of interest to assess publication bias. The distributions of data points provide limited evidence of publication bias in small studies (Figures 5–7).

## Discussion

Our study provides an important reference for clinicians in developing management and treatment plans of patients with LAM. The application of non-pharmacological therapies, especially physical therapy, not only improves their physical activity tolerance, but also may have a positive impact on their overall health status. This provides comprehensive options for patients with LAM and improves the diversity and personalization of treatment options.

Our study shows that interventions with multicomponent, including high intensity aerobic and strength training, yoga, pulmonary rehabilitation and telemonitoring exercise, are beneficial for patients with LAM. LAM is a multi-system disease that not only affects the lungs but can also have extrapulmonary manifestations. A diversified approach to non-pharmacological interventions addresses the varied needs of patients. High-intensity aerobic and strength training enhance cardiovascular and muscular functions, while yoga provides mental and physical balance (2); pulmonary rehabilitation targets respiratory function (3, 31), and telemonitoring ensures continuous engagement and adherence to treatment plans. By engaging in various structured and rigorous exercise regimens, patients can build their aerobic capacity and muscle strength, which are essential for maintaining daily activities and overall health.

Additionally, despite the included studies varied in intervention frequency and duration, all studies emphasized long-term sustained training. Given the chronic nature of LAM, ongoing management is crucial to maintain health and mitigate symptoms. Long-term training helps patients establish healthy habits, leading to sustained physical activity, and allows for gradual improvement in cardiovascular and muscular endurance, which is essential for managing LAM (32). Moreover, long-term programs offer regular support and motivation, enhancing patients' adherence (33). Regular customization and adjustment based on patient progress ensure effective and personalized interventions, optimizing patients' outcomes.

The low incidence of adverse events (5.7% overall dropout rate; no serious events) supports the safety profile of non-pharmacological therapies for LAM. The most common issues, transient dyspnea [Child (29)], muscle soreness [Araujo (28)], and joint discomfort [Li (30)], were managed through protocol adjustments (e.g., oxygen titration, intensity reduction). Notably, the telemonitoring approach demonstrated feasibility despite 14.3% dropout, suggesting real-world applicability with appropriate safety precautions (e.g., continuous SpO₂ monitoring). However, these findings should be interpreted considering risk mitigation strategies (as shown in 4 out of the 5 studies which implemented safety protocols, e.g., SpO₂>85% threshold), reporting limitations (e.g., retrospective studies, such as Gloeckl et al. (31), may underreport minor events), and special populations (e.g., patients with severe hypoxemia shown by FEV₁<30% were excluded in 3 studies). This safety profile further supports the clinical application of non-pharmacological therapies in LAM management, especially for outpatients and home-based intervention scenarios.

In the studies, participants' adherence and retention rates were high. High adherence and retention indicate that patients are willing to engage with and stick to the interventions, enhancing the reliability and sustainability of their treatment effects. High adherence and retention also reflect the acceptability and feasibility of the interventions, suggesting they can be successfully integrated into patients' daily lives, thus supporting wider adoption and application in clinical practice.

The meta-analysis results demonstrated that non-pharmacological therapies, particularly structured exercise programs, consistently enhance physical activity tolerance in patients with LAM. The improvements in 6-minute walk distance (6MWDT) across all studies, ranging from +37 m to +219 m, not only achieve statistical significance but also exceed the established minimal clinically important difference (MCID) of 30 m for interstitial lung diseases. This confirms that the observed improvements are clinically meaningful and can truly benefit patients' daily life. These findings align with Araujo et al.'s trial in LAM patients (28), showing similar functional benefits in chronic respiratory conditions. Importantly, enhanced physical tolerance translates to tangible clinical benefits: patients report better coping with daily activities, reduced dyspnea during routine tasks, improved independence, and greater psychological resilience and self-confidence, collectively contributing to comprehensive health improvements.

However, the impact on pulmonary function (FEV₁) and quality of life are not significant. Compared with other alveolar and parenchymal diseases (e.g., tuberculosis, chronic obstructive pulmonary disease, idiopathic pulmonary fibrosis) (34–36), LAM patients show similar improvements in exercise tolerance but less significant improvements in lung function, which may be related to the unique cystic lung pathology of LAM that is less reversible than other chronic respiratory diseases. Reasons for this may be further attributed to several interrelated factors: (1) The progressive nature of LAM, where advanced parenchymal damage may limit functional reversibility; (2) Potentially insufficient intervention duration or intensity to modify disease trajectory; (3) Heterogeneity in measurement tools for quality of life (e.g., SGRQ, SF-36, ATAQ-LAM) complicating cross-study comparisons; (4) Individual variations in disease severity and treatment adherence; and (5) Fundamental pathophysiological differences between functional capacity (6MWDT) and structural lung changes (FEV₁). These observations underscore the need for standardized outcome measures and adequately powered trials to clarify these relationships.

### Strengths

This study has some strengths. First, it provided important scientific evidence on non-pharmacological therapies for patients with LAM, especially in the application of physical therapy in the management of patients with LAM. These findings provide actionable guidance for a multidisciplinary care team, including: 1) Rehabilitation specialists to design progressive exercise regimens [e.g., aerobic-strength balance in Child (29)], 2) respiratory therapists to implement safe oxygen titration during activities, 3) nursing professionals to monitor daily functional status using 6MWDT, and 4) caregivers to facilitate home-based exercise adherence. By integrating these evidence-based strategies, teams can develop personalized LAM management plans that address functional and physiological dimensions of care. Second, findings of this study can be utilized by health care providers aiming to improve patients' self-management ability. By highlighting the effectiveness of non-pharmacological interventions, this study encourages patients with LAM to actively participate in their own health management. High-intensity exercise and other non-pharmacological interventions can help patients better control their health condition, improve their self-management skills, and thus improve their overall disease prognosis.

### Limitations

Although this study has some strengths, it still has limitations. First, it has a small sample size, which may lead to insufficient statistical efficacy to detect small but clinically meaningful effects, or increase the variability of the results, making the confidence interval of the pooled effects wide and affecting the statistical significance. Second, the study's scope of non-pharmacological therapies is limited. The existing literature mainly focused on physical therapy, such as high-intensity exercise, yoga, etc., while literatures about other non-pharmacological therapies (such as cognitive behavioral therapy, meditation, diet therapy, surgical therapy for recurrent pneumothorax, and nutritional support) are lacking. This may limit understanding of the overall effect of non-pharmacological therapies and may not fully reflect the potential benefits of non-drug therapy for patients with LAM. Third, most included studies had a follow-up time around 12 weeks (29) to one year (17), which may limit the assessment of the longer-term effects of the interventions. Given that LAM is a chronic progressive disease, its long-term outcomes may take more than one year to fully manifest, so the existing follow-up period may not be sufficient to observe the overall and long-term effect of the interventions. Lastly, some studies used a non-randomized controlled trial design (e.g., quasi-experimental study design or retrospective analysis design), which may lead to selection bias and other systematic biases that affect the accuracy of the results.

### Implication for future research and practice

This study provides directions for future research and important evidence for clinical practice. It is important to note the scarcity of studies on non-pharmacological therapies for patients with LAM, indicating a need for more research in this area in the future, especially studies with larger sample sizes and randomized controlled designs to reduce bias and improve result reliability. Additionally, the interventions reviewed primarily focused on physical therapies, such as high-intensity aerobic and strength training, yoga, pulmonary rehabilitation, and telemonitoring exercise. Future studies should explore other non-pharmacological therapies, such as diet therapy, psychological support (cognitive behavioral therapy, meditation), and surgical therapy for complications (e.g., recurrent pneumothorax), to provide a more comprehensive understanding of their potential benefits for patients with LAM. In clinical practice, health care providers can refer to the findings of this study to formulate personalized non-pharmacological intervention plans, emphasizing long-term sustained training and safety monitoring (e.g., SpO₂ monitoring) to improve patient adherence and outcomes. Furthermore, future studies should standardize outcome measures (especially for quality of life) to facilitate cross-study comparisons and meta-analyses.

## Conclusions

This systematic review and meta-analysis study not only demonstrates the potential value and safety of non-pharmacological therapies in the management of patients with LAM, particularly structured exercise programs that significantly improve physical activity tolerance (measured by 6MWD test), but also points the directions for future research. By further optimizing the interventions, expanding the sample size, standardizing outcome measures, and exploring a broader range of non-pharmacological therapies (e.g., diet therapy, psychological support), we can achieve a comprehensive understanding of the long-term effects of non-pharmacological therapies in patients with LAM, eventually providing more effective, personalized treatment options and better prognosis for patients.

## Data Availability

The original contributions presented in the study are included in the article/Supplementary Material, further inquiries can be directed to the corresponding author.

## References

[1] Khaddour K Sankari A Shayuk M. Lymphangioleiomyomatosis. In StatPearls. Treasure Island, FL: StatPearls Publish-ing (2024). Available online: Available online at: https://www.ncbi.nlm.nih.gov/books/NBK560686/ (Accessed June 1, 2024).

[2] Woodyard C. Exploring the therapeutic effects of yoga and its ability to increase quality of life. Int. J. Yoga. (2011) 4:49–54. 10.4103/0973-6131.85485 22022122 PMC3193654

[3] Spruit MA Singh SJ Garvey C ZuWallack R Nici L Rochester C. An official American thoracic society/European respiratory society statement: key concepts and advances in pulmonary rehabilitation. Am. J. Respir. Crit. Care Med. (2013) 188:e13–64. 10.1164/rccm.201309-1634ST 24127811

[4] McCormack FX Gupta N Finlay GR Young LR Taveira-DaSilva AM Glasgow CG. Official American thoracic society/Japanese respiratory society clinical practice guidelines: lymphangioleiomyomatosis diagnosis and management. Am. J. Respir. Crit. Care Med. (2016) 194:748–61. 10.1164/rccm.201607-1384ST 27628078 PMC5803656

[5] Johnson SR Cordier JF Lazor R Cottin V Costabel U Harari S. European Respiratory society guidelines for the diagnosis and management of lym-phangioleiomomatosis. Eur. Respir. J. (2010) 35:14–26. 10.1183/09031936.00076209 20044458

[6] Harknett EC Chang WYC Byrnes S Johnson J Lazor R Cohen MM. Use of variability in national and regional data to estimate the prevalence of lymphangioleiomomatosis. QJM. (2011) 104:971–9. 10.1093/qjmed/hcr116 21764810

[7] Cudzilo CJ Szczesniak RD Brody AS Rattan MS Krueger DA Bissler JJ. Lymphangioleiomyomatosis screening in women with tuberous sclero-sis. Chest. (2013) 144:578–85. 10.1378/chest.12-2813 23539171

[8] Yoon HY Kim HJ Song JW. Long-Term clinical course and outcomes in patients with lymphangioleiomyomatosis. Respir. Res. (2022) 23:158. 10.1186/s12931-022-02079-6 35717210 PMC9206248

[9] Peng JH Tu HP Hong CH. A population-based study to estimate survival and standardized mortality of tuberous sclerosis Complex (TSC) in Taiwan. Orphanet J. Rare Dis. (2021) 16:335. 10.1186/s13023-021-01974-3 34344419 PMC8330058

[10] Gupta N Finlay GA Kotloff RM Strange C Wilson KC Young LR. Lymphangioleiomyomatosis diagnosis and management: high-resolution chest computed tomography, transbronchial lung biopsy, and pleural disease management. Am. J. Respir. Crit. Care Med. (2017) 196:1337–48. 10.1164/rccm.201709-1965ST 29140122 PMC5694834

[11] Taveira-DaSilva AM Moss J. Clinical features, epidemiology, and therapy of lymphangioleiomyomatosis. Clin Epidemi-ol. (2015) 7:249–57. 10.2147/CLEP.S50780 PMC4396456 25897262

[12] Wang Q Luo M Xiang B Chen S Ji Y. The efficacy and safety of pharmacological treatments for lymphangioleiomyomato-sis. Respir. Res. (2020) 21:55. 10.1186/s12931-020-1316-3 32059669 PMC7023761

[13] Taveira-DaSilva AM Moss J. Management of lymphangioleiomyomatosis. F1000 Prime Rep. (2014) 6:116. 10.12703/P6-116 PMC4251421 25580270

[14] Yu J Henske EP. mTOR activation, lymphangiogenesis, and estrogen-mediated cell survival: the “perfect storm” of pro-metastatic factors in LAM pathogenesis. Lymphat. Res. Biol. (2010) 8:43–9. 10.1089/lrb.2009.0020 20235886 PMC2883473

[15] Gao N Zhang T Ji J Xu KF Tian X. The efficacy and adverse events of mTOR inhibitors in lymphangioleiomyomatosis: systematic review and meta-analysis. Orphanet J Rare Dis (2018) 13(1):134. 10.1186/s13023-018-0874-7 30107845 PMC6092843

[16] Barratt H Campbell M Moore L Zwarenstein M Bower P. Randomised controlled trials of complex interventions and large-scale transformation of services. Health Serv. Deliv. Res. (2016) 4(16). 10.3310/hsdr04160-19

[17] Lowder TW. High-Intensity exercise improves pulmonary function and exercise tolerance in a patient with TSC-LAM. Adv. Respir. Med. (2020) 88:356–9. 10.5603/ARM.a2020.0129 32869270

[18] Tufanaru C Munn Z Aromataris E Campbell JM Hopp L. Chapter 3: systematic reviews of effectiveness. In: Aromataris E Munn Z, editors. JBI Manual for Evidence Synthesis. Adelaide, Australia: JBI (2020). 10.46658/JBIMES-20-04

[19] Moola S Munn Z Tufanaru C Aromataris E Sears K Sfetcu R. Systematic reviews of etiology and risk, 2020. In: Aromataris E Lockwood C Porritt K Pilla B Jordan Z, editors. JBI Manual for Evidence Synthesis. Adelaide, Australia: JBI (2024). 10.46658/JBIMES-24-06

[20] Lockwood C Munn Z Porritt K. Qualitative research synthesis: methodological guidance for systematic reviewers utilizing meta-aggregation. Int. J. Evid.-Based Healthc. (2015) 13(3):179–87. 10.1097/XEB.0000000000000062 26262565

[21] Review Manager (RevMan) [Computer Program], Version 5.4; The Cochrane Collaboration: London, UK, 2020.

[22] Higgins JPT Thomas J Chandler J Cumpston M Li T Page M. Cochrane Handbook for Systematic Reviews of Interventions, Version 6.3. London, UK: Cochrane (2022). Available online: Available online at: https://training.cochrane.org/handbook (Accessed June 1, 2024).

[23] Melsen WG Bootsma MC Rovers MM Bonten MJ. The effects of clinical and statistical heterogeneity on the predictive values of results from meta-analyses. Clin Microbiol Infect. (2014) 20(2):123–9. 10.1111/1469-0691.12494 24320992

[24] Murad MH Wang Z Chu H Lin L. When continuous outcomes are measured using different scales: guide for meta-analysis and interpretation. Br Med J. (2019) 364:k4817. 10.1136/bmj.k4817 30670455 PMC6890471

[25] DerSimonian R Laird N. Meta-Analysis in clinical trials. Control Clin Trials. (1986) 7:177–88. 10.1016/0197-2456(86)90046-2 3802833

[26] Bell ML Fiero MH Dhillon HM Bray VJ Vardy JL. Statistical controversies in cancer research: using standardized effect size graphs to enhance interpretability of cancer-related clinical trials with patient-reported outcomes. Ann Oncol. (2017) 28(8):1730–3. 10.1093/annonc/mdx064 28327975 PMC5834129

[27] Page MJ McKenzie JE Bossuyt PM Boutron I Hoffmann TC Mulrow CD. The PRISMA 2020 statement: an updated guideline for reporting systematic Re-views. Br Med J. (2021) 372:n71. 10.1136/bmj.n71 33782057 PMC8005924

[28] Araujo MS Baldi BG Freitas CSG Albuquerque ALP Marques da Silva CCB Kairalla RA. Pulmonary rehabilitation in lymphangioleiomyomatosis: a controlled clinical trial. Eur. Respir. J. (2016) 47:1452–60. 10.1183/13993003.01683-2015 26917604

[29] Child CE Kelly ML Sizelove H Garvin M Guilliams J Kim P. A remote monitoring-enabled home exercise prescription for patients with interstitial lung disease at risk for exercise-induced desaturation. Respir. Med. (2023) 218:107397. 10.1016/j.rmed.2023.107397 37640274

[30] Li X Xu W Zhang L Zu Y Li Y Yang Y. Effects of yoga on exercise capacity in patients with lymphangioleiomyomatosis: a nonrandom-ized controlled study. Orphanet J. Rare Dis. (2020) 15:72. 10.1186/s13023-020-1344-6 32178705 PMC7075042

[31] Gloeckl R Nell C Schneeberger T Jarosch I Boensch M Watz H. Benefits of pulmonary rehabilitation in patients with advanced lymphangioleiomy-omatosis (LAM) compared with COPD: a retrospective analysis. Orphanet J. Rare Dis. (2020) 15:255. 10.1186/s13023-020-01540-3 32962746 PMC7507679

[32] Puhan M Scharplatz M Troosters T Walters EH Steurer J Group CA. Pulmonary rehabilitation following exacerbations of chronic obstructive pulmonary disease. Sao Paulo Med J. (2010) 127(5):322. 10.1590/S1516-31802009000500016

[33] Haskell WL Lee IM Pate RR Powell KE Blair SN Franklin BA. Physical activity and public health: updated recommendation for adults from the American college of sports medicine and the American Heart Association. Med. Sci. Sports Exerc. (2007) 39:1423–34. 10.1249/mss.0b013e3180616b27 17762377

[34] McCarthy B Casey D Devane D Murphy K Murphy E Lacasse Y. Pulmonary rehabilitation for chronic obstructive pulmonary disease. Cochrane Database Syst Rev. (2015) 2015(2):CD003793. 10.1002/14651858.CD003793.pub3 25705944 PMC10008021

[35] Dowman L Hill CJ May A Holland AE. Pulmonary rehabilitation for interstitial lung disease. Cochrane Database Syst Rev. (2021) 2(2):CD006322. 10.1002/14651858.CD006322.pub4 34559419 PMC8094410

[36] Visca D Zampogna E Sotgiu G Centis R Saderi L D’Ambrosio L. Pulmonary rehabilitation is effective in patients with tuberculosis pulmonary sequelae. Eur Respir J. (2019) 53(3):1802184. 10.1183/13993003.02184-2018 30872556

